# “I just think that we should be informed” a qualitative study of family involvement in advance care planning in nursing homes

**DOI:** 10.1186/s12910-016-0156-7

**Published:** 2016-11-10

**Authors:** Lisbeth Thoresen, Lillian Lillemoen

**Affiliations:** 1 Centre for Medical Ethics and Department of Health Sciences, Institute of Health and Society, University of Oslo, Postboks 1130, Blindern, 0318 Oslo, Norway; 2 Centre for Medical Ethics, Institute of Health and Society, University of Oslo, Postboks 1130, Blindern, 0318 Oslo, Norway

**Keywords:** Advance Care Planning, Qualitative study, Autonomy, Family ethics

## Abstract

**Background:**

As part of the research project “End-of-life Communication in Nursing Homes. Patient Preferences and Participation”, we have studied how Advance Care Planning (ACP) is carried out in eight Norwegian nursing homes. The concept of ACP is a process for improving patient autonomy and communication in the context of progressive illness, anticipated deterioration and end-of-life care. While an individualistic autonomy based attitude is at the fore in most studies on ACP, there is a lack of empirical studies on how family members’ participation and involvement in ACP- conversations may promote nursing home patients’ participation in decisions on future treatment and end-of-life care. Based on empirical data and family ethics perspectives, the purpose of this study is to add insights to the complexity of ACP-conversations and illuminate how a family ethics perspective may improve the quality of the ACP and promote nursing home patients’ participation in advance care planning.

**Methods:**

Participant observations of ACP-conversations in eight nursing homes. The observations were followed by interviews with patients and relatives together on how they experienced being part of the conversation, and expressing their views on future medical treatment, hospitalization and end-of-life issues.

**Results:**

We found that the way nursing home patients and relatives are connected and related to each other, constitutes an intertwined unit. Further, we found that relatives’ involvement and participation in ACP- conversations is significant to uncover, and give the nursing home staff insight into, what is important in the nursing home patient’s life at the time. The third analytical theme is patients’ and relatives’ shared experiences of the dying and death of others. Drawing on past experiences can be a way of introducing or talking about death.

**Conclusions:**

An individual autonomy approach in advance care planning should be complemented with a family ethics approach. To be open to family ethics when planning for the patient’s future in the nursing home is to be open to diversity and nuances and to the significance of the patient’s former life and experiences.

## Background

As part of a larger research project on end-of-life communication in Norwegian nursing homes, we have carried out a qualitative study on Advance Care Planning (ACP) conversations between nursing home staff, nursing home patients and relatives. The concept of ACP is internationally accepted as a process for improving patient autonomy and communication in the context of progressive illness, anticipated deterioration and end-of-life care [1, 2], also in nursing homes [3]. Based on empirical findings from observations of ACP-conversations in Norwegian nursing homes, followed by interviews with the patients and their relatives, in this article we look at, and elaborate on, how family involvement may improve the quality of the ACP-process and even promote nursing home patients’ participation in advance care planning. We present empirical data that illuminates how nursing home patients and their relatives are involved in each others lives, even if their everyday lives are separate. What we see as close family relations and patients’ and relatives’ shared life stories, have made us reflect on whether patient autonomy and self-determination are sufficient ethical values to underpin advance care planning, especially when regarding frail nursing home patients.

Dying patients’ legal and ethical rights to autonomy, and to be informed and make decisions are often described as central characteristics of modern, individualized end-of-life care [4–6]. Families are given increased attention, for instance as the target for, and part of hospice care [7]. Nevertheless, Levine [8] claims that families’ participation in health care is more complex these days because of the aging population, the fact that more (technological) care is provided at home, and new family structures. Levine points to the need to improve informal caregivers’ well-being through better communication and involvement in the care of their loved ones. In analyzing the development of end-of-life care in the US, Wolf et al [9] describe three phases: “Securing rights (1976-1994)”, “Facing clinical realities (1995-2009),” and “Reforming end-of-life care systems (2010-).” In the last and ongoing phase, the need to include friends and families in end-of-life decisions is highlighted [9], and this development in bioethics [8] is also addressed in guidelines on end-of-life care [10]. Among others, Hilde Lindemann (Nelson), and James Nelson have contributed with important insights into families as moral communities and the importance of family perspectives in medical decision-making [8, 11, 12]. Beside individualism, impartiality and universalizability as common themes in contemporary ethical theories, the authors describe collectivity, favoritism, particularity, nonconsensuality and pre-modern sensitivity as characteristics of what is called the morality of intimacy [12]. *Collectivity* is connected to the ongoing and important process of group self-definition in families, and *favoritism* indicates the value of being loved for your self. *Particularity* means to pay attention to the small details of everyday family routines and shared meaning, and how this can promote feelings of solidarity. *Nonconsensus* covers questions concerning where the duty to care for family members comes from. *Pre-modern sensitivity* pays attention to the special moral significance of families, and how to develop family ethics ‘navigation aids’ or ‘stars.’ Such navigating ‘stars’ in ‘the ethical landscape’ [12] (p. 73-74) represent that “family members are stuck with each other” and “families are ongoing stories” [12] (p. 74). According to the authors, the navigating stars are not ethical rules or guides for conduct, but should work as reminders for health care professionals that family matters morally. A family ethics approach pays attention to all family members, and autonomy is considered to be relational. Lindemann describes families as “nested in a web of identity-constituting relationships” [11] (p.352) and how the patients’ decisions and choices are influenced by this.

Family matters can be understood morally in two ways: *the instrumental* value of families; that is how families may look after each other or care for each other in different ways, and *intrinsic* value; that “families are also valuable ends in themselves” [11, 13] ([11] p.18).

We find that perspectives from family ethics are not often taken into account in ACP research, even if many studies pay attention to families’ impact on ACP, and the importance of trust and reliability between patient and relatives [14–17]. The main focus in the literature seems to be on the patient’s right to be informed and take part in decisions concerning medical treatment and end-of-life care [3, 14, 15, 18–20]. In the present study, theory on family ethics has been useful to sensitize our understanding of the complexity of ACP-conversations, and we ask whether family ethics perspectives may strengthen the nursing home patients’ possibilities to get his or her values and preferences known or expressed. We also add valuable insight into *how* advance care planning is carried out. Even if end-of-life communication has been studied for many years, this perspective is lacking [21–25]. Our main research questions are: how can family involvement in ACP-conversations 1) contribute to identifying what is important to the nursing home patient at this point of life, and 2) promote the patients’ participation in decisions on future treatment and end-of-life care?

### The Norwegian health care system and legislation

The Norwegian health care system is divided between the primary sector (primary health and long-term care) and the secondary sector (hospitals and specialist services), and is based on the Scandinavian Welfare Model. This means that health care services are primarily financed and provided within the public sector [26]. Health care for the aging population, such as home care and nursing homes, is mainly delivered by the municipalities, and living at home for as long as possible has been a clear political vision for many decades [27]. This might explain why the nursing home population is characterized by high age (mean age is 84), vulnerability, disability and multiple diagnoses [28, 29]. 47 % of the Norwegian population ends their life in a nursing home (numbers from 2014) [30]. 31 % of the population dies in hospitals, while 14 % die at home [30]. The high number of nursing home deaths makes this a particularly relevant place for carrying out research on decision-making and family ethics. However, nursing home patients with cognitive impairment make conversations about future medical treatment difficult for all parties involved [24, 29, 31, 32]. This indicates the importance of early timing of conversations about the future, and the importance of documenting patients’ views, wills and preferences to help future decision making [33]. The legal right for patients to be informed and participate in health care decisions is established by the Patients’ and Users’ Rights Act and especially chapter 3 (The right to information and participation) and chapter 4 (Consent to health care). Section 4-9 says that “A dying patient is allowed to reject life-prolonging treatment.” If the patient is not able to communicate his or her wishes, “information may be obtained from the patient’s next of kin in order to determine what the patient would have wanted (Section 4-6) [34]. Health care staff is responsible for assessing patients’ competence to consent, which requires sufficient moral reflection, knowledge and communication skills [35]. A Norwegian guideline on decision-making processes in the limitation of life-prolonging treatment [33] recommends ACP-conversations as a way of involving patients in questions concerning treatment at the end of life. In the guideline, ACP is described as a voluntary communication process, where the purpose is to help the patients to reflect on their goals, values and beliefs, to articulate wishes and preferences for future end-of-life care and medical treatment [1, 36] and to name their proxy in case of future acute or permanent inability to give consent [37]. Euthanasia and assisted suicide are illegal in Norway, and living wills are thus not legally binding [36].

## Methods

The present study is part of a larger research project at the Centre for Medical Ethics at the University of Oslo; “End-of-life Communication in Nursing Homes. Patient Preferences and Participation”, which aims to map, examine and enhance the existing practices and routines for advance care planning in Norwegian nursing homes. The larger research project consists of three parts, with different methods and perspectives. Part one is a literature review on implementation and research strategies of ACP in nursing homes [38]. In part two we study if and how ACP is used in Norwegian nursing homes and this part consists of A) a survey to investigate practice and prevalence of ACP in Norwegian nursing homes *and* B) a qualitative study of ongoing advance care planning practices in nursing homes. In the qualitative study, we wanted to explore how ACP is actually carried out, and the experiences of those participating in such conversations. Part one and two have contributed to identifying routines, habits and experiences in nursing homes which we could copy or learn from in part three of the research project. This third part consists of developing, implementing and evaluating a guide for ACP-conversations in nursing homes. See Table 1 for an overview of the research project and the three parts.

**Table 1:** Overview research project

Research project: end-of-life communication in nursing homes. Patient preferences and participation	
Part 1 Literature review on implementation and research strategies of ACP in nursing homes	Part 3 Developing, implementing and evaluating a guide for ACP in nursing homes
Part 2 A) Survey to all Norwegian nursing homes to investigate practice and prevalence of ACP B) Qualitative study in 8 nursing home wards to explore how ACP is carried out and experiences with participating in ACP	

This article presents findings from the qualitative study (B, part two) on how ACP conversations are practiced and experienced, paying special attention to empirical findings on families’ impact and participation in ACP (for other results from the qualitative study, see Thoresen et al. [39]).

### Participants

The qualitative study was carried out in the spring of 2014, and the first author (LT) was responsible for recruiting nursing homes as well as conducting observations and interviews. The plan was to recruit 6-8 nursing homes, and in the end 8 nursing home wards accepted our invitation to participate in the research study. This means that we were allowed to observe an ACP-conversation between nursing home staff, patients and/or relatives, and conduct interviews with the participants. The wards were recruited through networks, Facebook, mailing lists and ‘spreading the word’ in conferences and seminars. None of the eight nursing home wards fully answered the four recruitment criteria. These were: 1. Established and practiced ACP- conversations for at least one year, 2. Uses a list of prepared questions in the conversations, 3. Includes patients with dementia, and 4. The nursing home physician takes part in the conversations. On each nursing home ward, contact was established with one staff member (nurse or physician) who helped with local arrangements. In the nursing homes, advance care planning was often part of admission conversations or similar routinely held conversations, and when such conversations were planned, the local contact assessed whether this was a conversation suitable for observation followed by interviews. Detailed information about who participated in the conversations, what the conversation was called, place and length can be found in Table 2.

**Table 2:** Detailed information on ACP-conversations and participants

Nursing home	Conversation term	Patient	Relatives present	Staff present	Place and length	Template for ACP questions?
A	Admission conversation	Woman, 91, stroke, aphasia, in wheelchair. Married.	Daughter	Physician, primary nurse, head nurse	Duty room 30 minutes	Yes
B	Admission conversation	Woman, 89, wheelchair, amputee leg, blind. Widow.	Daughter	Physician, nursing assistant, two nurses, head nurse	Duty room 30 minutes	Yes
C	Preparatory conversation Routinely held when patient was diagnosed as dying.	Woman, not present, 90, severe dementia, married.	Husband, son, daughter.	Physician, nurse	Meeting room, 45 minutes	Yes
D	Bi- annual conversation	Man, 80, stroke, wheelchair, married	Wife	Physician	Patient’s room 25-30 minutes	No
E	Admission conversation	Woman, 87, early dementia, seems healthy, single?	Niece	Physician, intern, primary nurse	Living room, 50 minutes	No
F	Bi-annual conversation	Woman, 88, stroke, aphasia, widow	Son	Physician, nurse, nurse student	Living room, 30 minutes	No
G	No observation					
H	Admission conversation	Woman, 100, early dementia, severe hearing loss	Daughter, son in law	Physician, nurse, head nurse	Patient’s room, 70 minutes	Yes

### Participant observations

Participant observation is a qualitative method which aims to bring impressions and rich data of how people interact in particular social situations and contexts. Direct observations bring other insights than interviews, and “sharing” situations with informants make follow-up interviews far more targeted [40, 41]. LT conducted participant observations of seven ACP-conversations between nursing home staff, patients and relatives. Even though an observation was planned in nursing home G, when LT arrived at the nursing home, this was not possible, and instead two relatives, a physician and a nurse agreed to be interviewed together. The observed conversations were carried out in the patients’ room, in the living room or in the duty room; sometimes the participants sat around a table; sometimes in a circle or in a more informal manner. During each observed conversation, LT took notes on the room and how the participants were placed, what happened during the encounter, who was talking and about what, participants’ facial and bodily expressions, movements, and the atmosphere. Verbal questions and answers were written down verbatim. It was important to take time shortly after the observation to go through, expand and reflect on the observation notes.

### Qualitative interviews

Qualitative interview is a well-suited method when the aim is to get to know how the informants describe their experience [42], in this case of taking part in advance care planning. All interviews took place immediately after the ACP-conversations, except for an interview with staff in nursing home H, and an interview with a daughter in nursing home B which, for practical reasons, was carried out a few days later. In nursing home D, circumstances led LT to decide not to interview the resident and his wife. The conducted interviews can be described as conversations with a structure (interview guide) and a purpose [42]. Interview questions covered issues like how patient and relatives were informed about and invited to the meeting; what they thought the aim of the meeting was and whether they knew what was on the agenda; whether patient and relatives had ever talked with each other about treatment, dying and death (prior to the meeting); how they experienced discussing these issues; whether they wanted the conversation to be different in any way, and so on. The interview also centered on the observed conversation and what had taken place. The interviews with patient and/or relative lasted from 15 to 45 minutes. Interviews were tape recorded and transcribed verbatim by LT and a master’s student.

### Analysis

The method of content analysis was useful in analyzing the interviews, since we wanted to focus on aspects related to the research questions [43]. This means that we searched for how patients and relatives answered questions concerning their experience of participating in the conversations; how they were invited and informed about the conversation; whether they had talked about end-of-life care earlier and so on. We have also carried out a thematic coding of the data material where nuanced aspects of how ACP matters to families, but also how relatives may contribute to improved ACP-outcomes, came to fore. We became aware of how strongly planning for the future belonged, not only to the patient, but also to the (whole) family who talked about their responsibilities towards their loved ones. This made us develop more precise research questions to the material, presented in the introductory part of the article: how can family involvement in ACP-conversations 1) contribute to identifying what is important to the nursing home patient at this point of life, and 2) promote the patients’ participation in decisions on future treatment and end-of-life care? These research questions, as well as analytical findings, were also influenced by the participant observations where LT was left with questions about what was going on and why [44, 45]. Perhaps observations of how frail, elderly patients struggled to answer questions about their (unsure) future, lead to what Gubrium and Holstein [45] describes as a tentative insight; an insight that continues to influence the research process and which works as an analytic inspiration. In this case, the ‘insight’ made us want to understand better the patients’ autonomy and self-determination in the ACP-conversations, and also the role of the family members. The research questions are also informed by theoretical perspectives from family ethics which means that the findings are both inductively and deductively informed.

### Ethical considerations

A main ethical principle in research studies involving people is informed and freely given consent to take part in the study [46]. Staff, patients and relatives were informed about the study in writing and orally by the local contact person, and asked if they wanted to participate. Everyone who was invited agreed to participate. This was also the case with staff members, and informed, written consent was gained from all participants. In nursing home C, the conversation was conducted without the patient because she was too ill to participate. When patients are recruited by the nursing home physician or nurses who they are totally dependent on, questions of how freely consent is given have to be taken into consideration. Patients with dementia who were not able to give consent were excluded from the qualitative study. However, two of the patients were described as being in the early phase of dementia, and two of them were not able to speak because of aphasia. According to the nursing home staff as well as the relatives, the latter two were cognitively intact, but it was difficult to be sure that they fully understood written and oral information about the project. Even if the researcher is responsible for assessing and being careful in cases like this, we found that we could trust the relatives and staff when they said that each of the two residents were able and willing to take part in the study. It is important to include people who are vulnerable and perhaps not able to speak for themselves in research studies, in order to gain some insight into their lives and experiences [47]. Based on written and oral information, all participants gave their written consent. Nursing home residents who lacked consent capacity could not take part in the study, and the local contact person was responsible for making these assessments.

Being observed by researchers can be experienced as intrusive and degrading, and especially when talking about deteriorating health and death, which are sensitive and private matters to many [48]. Even if the aim of the observation is described as getting an impression of the content of conversations and how they are carried out, staff can easily think that they are being evaluated by the researcher [49], and residents/relatives can find it difficult to understand what the researcher actually does.

Permission to carry out observations and interviews was granted from the head of the nursing homes and from NSD which is the Data Protection Official for the University of Oslo (NSD 37368). When applying to the Regional committee for medical and health research ethics (REC), the answer was that the research project did not require approval from REC (2013/19937).

## Results

In this section we will present what we see as the main results. The three close thematic findings show how, in ACP-conversations, 1. Patients and relatives are perceived as an intertwined unit, 2. Relatives’ involvement and participation in ACP-conversations is significant to get to know what is important in the patients’ lives, and 3. Families share experiences of dying and death. Before continuing to present the results in detail, we want to add some information about the conversations. In the participating nursing homes, questions concerning future medical treatment and end-of-life care were raised as part of admission conversations and bi-annual routine conversations. Only in nursing home C was the sole aim of the conversation to talk about and discuss future medical treatment and withdrawal of treatment. The patients who took part in the conversations were asked about DNR, if they wanted medical treatment in case of infections like pneumonia, if they wanted hospitalization and if they had any particular thoughts or wishes about dying and death. Only in one case was the patient asked a ‘non-medical’ question; if he had any hopes or worries for the future. Even if there were one or more nurses present, it was always the physician who raised the ACP-questions.

### Relatives and patients; an intertwined unit

All the patients who participated in our study were (more or less) competent. This should indicate that it was possible to conduct a conversation about health issues and future treatment with patients on their own, but in all of the observed conversations one or more (close) relatives were present. A relative is defined in the Patients’ and Users’ Right Act (§ 1-3) as the person or next of kin who the patient notifies as his/her relative. In our study, six daughters, two sons, two spouses, one son-in-law and one niece took part as relatives in the encounters with the nursing home staff. Thus, all the relatives were close family members, and there were no friends of the patients among the relatives. Some nursing homes had written information and procedures on how ACP-conversations between patients, relatives and staff should be carried out, what the aims of the meetings were, and who should participate. In these documents, nursing home patients and relatives were more or less treated like one unit, because they were often mentioned together or addressed together. For instance, nursing homes held admission conversations where questions about future medical treatment were raised, called *Physician, patient and relatives’ conversation,* or *User and relatives’ conversation.* As already described, observing the conversations confirmed the impression of patients and relatives being connected and intertwined, and of how the patient’s well-being was related to their relationship to their loved ones.

The observation of a conversation between Arne, who has lived on the nursing home ward for more than five years, his wife Liv who visits Arne every day, and the nursing home physician illustrates the point of being intertwined. The conversation took place in Arne’s room and centered on the present unstable medical situation, possible new strokes and whether Arne needs hospital admission in case of a new stroke. The physician wonders if it is better for Arne to stay on the ward, and he asks Liv what she thinks about hospital admissions. They remind each other that the last pneumonia was well taken care of in the nursing home, and both Liv and Arne agree that in case of a new stroke, the hospital should be consulted before any decision about admission is made. The physician then asks Arne: “Do you have any other worries for the future – or hopes?” Arne refers to a documentary about a wheelchair bound man who trained himself to walk, and asks if that could possibly happen (to him)? Liv comments on this as a wishful dream, and the physician states that this is not possible for Arne. Liv continues by telling us that a density has been found in one of her lungs, and that she needs to go through further examinations. The physician asks questions concerning her symptoms, and while Liv’s possible illness is talked about, the atmosphere in the room is felt by LT as becoming tense. Arne says that he tries not to think about how Liv may be ill, and that everything is going to be fine. The physician says that the staff should be informed because Liv visits every day, and if she is ill, they should know.

We find that this case illustrates well that issues concerning the aging patients’ deteriorating health, what is important today, and what may happen in the future is something that not only ‘belongs’ to the patient. These are issues that are also part of the relatives’ daily life as well as the rest of the family. What Arne wants for himself is closely related to Liv’s former experiences and knowledge about Arne, and as this case shows, what happens to Liv means a lot, perhaps everything to Arne. The couples’ everyday life is intertwined in many ways, and it is difficult to see how Arne could plan for the future without consulting or involving his wife, and the other way around. Liv and Arne are involved with and dependent upon each other.

In one of the nursing homes, the physician’s practice was quite the opposite. Prior to admission conversations where the relatives were invited, the physician always talked to the patients alone about their future wishes and preferences. The physician explained that it was important to talk to patients on their own, to be sure that the patients’ wishes and preferences were not affected by what relatives thought. This means that during the observed admission conversation, where the patient and her daughter were present, as well as the physician and other staff, the physician informed all of us of what the patient had already expressed as her wishes and preferences on future medical treatment and hospitalization. In the interview some days later, the daughter said that she wished she had been informed in advance about the dialogue between her mother and the nursing home physician and she said: “I find it to be a good thing that they want to talk to her, because she is able to express her views. But, I wish we could have talked to each other in advance, and I also would have liked to talk to my brother. I just think that we should have been informed.” The daughter told us that her mother had recently been through a very tough time where she lost a leg and now she was bound to a wheelchair. The daughter thought that her mother should have been spared being asked questions about wishes concerning future medical treatment and end-of-life shortly after this extremely hard period. We find that this example highlights how the daughter felt that she was somewhat set aside by the physician. She had followed her mother closely for many years, and had recently faced a hard time by her side. In the interview, the daughter repeated many times that at the one hand, it was right to ask her mother what she wanted, but on the other hand, she expressed that it was also kind of harmful to her to have to answer the questions. The daughter was fully aware of the value of autonomy, but she also wanted to protect her mother. Another relevant issue in this particular nursing home is that the daughter was informed about her mother’s preferences along with the staff. This way, she was treated like the staff, even though she had very different knowledge about, and relation to the patient. What we see as sensitive issues concerning future illness and dying is handled as information in an efficient way. During the observed admission conversation, there was no room for reflections on the impact these questions had on the patient or the daughter.

The daughter above, as well as a niece in one of the other nursing homes, expressed what we interpret as responsibility and protectiveness towards their loved ones. In the case with the niece, she participated in the ACP-conversation along with her aunt. The aunt seemed to struggle to answer questions concerning future illness, whether she wanted to be resuscitated if her heart stopped and whether she had thoughts about dying and death. In the interview, the niece told us that she wished she had had the chance to talk to her aunt before the conversation, so that her aunt was better prepared for the serious questions concerning medical treatment and end-of-life care. It was not that the niece didn’t want her aunt to have the ACP-conversation; she only wished that they had talked about it first.

As a close relative, you may not have any other option than being by the side of, for instance, your old mother. In the observed conversation in one of the nursing homes, the participating daughter was the patient’s – Asta’s - only child. Because of aphasia, Asta couldn’t speak, and her daughter had to answer questions on her behalf. During the conversation, the daughter held Asta’s hand, and she repeated the physician’s questions to Asta, using some other words. Their faces were near, and it looked like a warm and trusting relationship. When the physician asked about DNR, they both quietly started to cry. In the interview after the conversation, the daughter denied that it was difficult to know what to answer as a proxy. She said that she and her mother had never talked directly about future medical treatment or end-of-life issues, but “I know her so well, I know what matters to her.”

Our data shows that planning for the future is something that relatives want to be involved in. One daughter expressed that she wished that she could have consulted her brother about the medical questions that were brought up during the ACP-conversation, and one son told us that he represented his two brothers in meetings such as this conversation. He often discussed issues related to his mother with his brothers, and then “they send me because I can come here during the daytime.” In the interview in nursing home G, two sisters talked about the importance of being able to talk and discuss with each other during the time of their father’s weakening and finally death.

### Relatives’ involvement and participation in ACP-conversations is significant to get to know what is important in the nursing home patients’ lives

As described in the literature, an ACP-conversation should cover more than mainly medical questions. Getting to know what is important to the patient today, and what are perhaps future worries is vital to be able to understand and plan for end-of-life care. Analyzing field notes of the conversations and interviews with patients and relatives through the lenses of a family ethics perspective, we became aware of what we interpret as a mismatch between what matters to the patients and what the focus of the conversations are.

One of the conversations illustrates this well. Jenny had just moved onto the ward and prior to the ACP-meeting, the nurse and Jenny had exchanged information about Jenny’s former life, and about daily routines on the nursing home ward. The nurse started the conversation by sharing with us what Jenny had answered to different questions about her life. Jenny herself was listening quietly during this part. When the physician took over, and shifted to questions concerning future medical care, hospital admission, and also asked Jenny if she had any thoughts or wishes concerning the dying phase, she replied: “It is difficult to answer (your question),” or: “I haven’t thought about it, but I don’t think one should start (DNR),” or she didn’t answer at all, looking like she wanted to excuse herself. All in all, Jenny’s participation in the conversation was perceived (by LT) as quiet, passive and hesitant. At the same time, Jenny seemed to follow intently what was said, and her facial expression and bodily movement made her look tense. In the interview with Jenny and her niece that followed straight after the conversation, the impression of Jenny as a perhaps shy or quiet person changed to some extent. LT asked Jenny how she felt about taking part in the conversation, and whether she had ever talked with anyone about dying and death. When Jenny said that she hadn’t talked to anyone about such things before, her niece asked: “Not even with Brita, your sister?” Jenny answered no once more, and then continued to tell us about her sister who died just a few months ago: “We have always been together, we grew up together, we worked together and we lived together; we have had everything together.” The niece continued: “But the two of you never talked about death, not even when she became ill?” “No, (Jenny continues), we have been a little…not so communicative about such things.” Then she tells us about a friend who is coming to visit.

The patient Mary had also just been through severe losses; her sister died a year ago; they were close. Then she lost a leg and now she had lost her home because she had to move to a nursing home after the amputation. This affects Mary’s wellbeing and mood, her daughter told us during the interview: “She (Mary) has lived at home until now, and her hope was to continue to stay at home as long as possible, and then all of a sudden, everything is changed, and of course, that makes you lose your strength and your optimism, and…it doesn’t help (your mood) to have such a conversation (ACP-conversation) right after admission to the nursing home…I can see that she has become …you become a bit depressed moving to a nursing home because it is the last stop.” The dramatic changes in Mary’s life were not mentioned in the observed ACP-conversation, except from some brief information from the physician about the amputated leg. While the daughter is able to connect the dramatic changes in her mother’s life with the ACP questions raised by the physician, this connection is not mentioned by any of the staff during the ACP-conversation.

In the interview with the patient Asta and her daughter after the ACP-conversation, the daughter told us that the biggest problem for Asta is that since she moved to the nursing home, Asta and her husband had to live separated lives: “And my old father, he is 95 and he wonders when she (Asta) is coming back home. If he wants to come here, he has to come by taxi…sometimes he only comes Saturday and Sunday.” What the daughter described as her parents’, as well as her own, biggest problem was not brought up in the admission conversation about Asta’s life and future in the nursing home. Perhaps Asta’s daughter is right when she claimed that: “(the conversation) was obviously meant to be about medical issues…the focus was on the medical part.”

We find that the mismatch between which questions and issues are at stake in the conversations and what came to fore in the interviews underlines the importance of a family perspective in advance care planning. To Jenny, Mary and Asta, their close relatives could have added important and relevant information in the ACP-conversations if other kinds of questions had been asked. They knew their life stories, what had recently happened, and what matters to their family members today.

### The families’ shared experiences of dying and death

This finding is closely related to the former one, where the relatives’ knowledge of the patient’s life can bring to fore important issues to be talked about in ACP-conversations.

For many people, dying and death is hard to talk about. The participants in our study were no exception; both health care staff as well as relatives expressed that they found it difficult to talk about or raise questions concerning the future death of the patient/family member. The observations showed that it was the physician who was responsible for turning the conversation in the direction of treatment choices and death. When asked in the interview how they experienced raising these questions, more or less all physicians described this as challenging. However, they conducted conversations like this routinely, and had been through similar meetings many times. For most patients and relatives it was quite the opposite, this was the first time they took part in a conversation like this. When they were asked how they experienced talking about treatment choices and dying, one son answered: “… (the conversation) introduced the topic of death, and we are not particularly good at talking about it – it is not a common topic of conversation in our family…we have talked very little on a deeper level”. A daughter expressed in the interview, that to be honest, she hadn’t thought much about the future death of her mother, and she had never talked with her mother about dying or death. This is similar to another son who confirmed that he had never touched upon these issues with his mother before she became ill. He added that he found it important, but very difficult, to have the conversation with the nursing home physician. These answers were representative for nearly all the relatives. When we asked them if the patient had ever talked about, expressed any wishes or made any kind of plans for their last phase of life, the answer was mostly no; one’s own death was not talked about nor planned for in any way. None of the relatives or patients could tell us about proper conversations in the family about dying and death, nor the patients’ future death.

However, what was talked about and shared in the families (we were told in the interviews) was the death of others. That is; how people close to the family or family members had died; either in accidents or because of serious illness, the impact of dying and deaths on the family, how the funerals had been and the sad memories. For instance, when Asta’s daughter was asked if she and her mother had talked about death before the ACP-conversation, she answered after a long time: “…No, let’s see – we have talked a little about the funeral, and my father, he wants to be buried, which is different from the rest of the family who want to be …and then, our nephew was in this terrible accident, and is also cremated – but except from that, we haven’t talked about it. No, we haven’t. …we try to see it as natural as possible; we are all going away from here. We have talked about those that are left behind, and my parents have been so healthy and many of their younger friends have died. It is sad to think about. And we have talked about the sadness of dying – and to older people, it is sad, but natural to die”.

A son also pointed to the deaths of other people as the link to his mother’s death: “She had a stroke before, and she has recovered more or less, and then we (the son and his mother) talked about other people in the same situation…and we had some experiences with dad who died in this nursing home…we learned a lot during his last days. So we were prepared when mother moved in to the ward.” A niece expressed: “I have thought a lot about all the losses old people experience, you (addressing her aunt) have lost so many friends.” The niece and her aunt had also experienced the death of a fellow relative in the same nursing home.

All the patients and relatives who participated in our study had experienced serious illness, dying and deaths of family members, friends or people they knew. They had visited dying people, and they may have reflected upon the weakening and fading bodily process of dying. They had been to (many) funerals and they had experienced grief. Talking about dying and death is described as difficult in Western culture, and as we can see in our data, the future dying of older family members is not talked about inside the family. But even if family members don’t talk to each other, we claim the importance of paying attention to how people have experienced the death of others; how they ‘practice’ around the death of others, and how shared experiences and memories about former deaths may be part of the bigger family narrative. Indeed, we find this to be relevant when planning and talking about one’s own future and end of life.

## Discussion

Patient involvement in health care decisions has been addressed in Norwegian White papers for many years [50–52]. To improve user involvement, patients should be asked what is important to them now and in the future [50], and patients’ preferences and wishes must be taken into account when planning for health care and treatment [52]. In the nursing homes in our study, the staff tried to actualize these values by practicing advance care planning. However, after observing ACP-conversations, we are left with the impression that patients only to a limited extent were able to fully understand what was taking place, or express their views. Even if the principle of autonomy was realized, as well as their legal right to be informed about, and participate in decisions, the patients’ capacity to participate was limited [53]. We agree that end-of-life care and end-of-life care decisions should be in line with the patients’ wishes and preferences, and we wonder if a family ethics approach could help improve our understanding of what that means. In this section, we discuss how family ethics can be understood as part of a development where the moral implications of relations are given greater attention. We then point to the danger of ‘family paternalism,’ before discussing what the aims of an ACP-conversation should be: factual outcomes and/or a family-centered process?

### The moral impact of relations

Even if married couples like Liv and Arne may be involved in each other lives in a special way, in our study, we see the same tendency in the way children and a niece are tied to their parents or an aunt, and because of this, also want to be involved in planning for the future. In family ethics, this involvement is taken for granted or is described as a *give*n part of being in a family. Families are supposed to care for each other, make decisions and act in each other’s best interest [13]. We understand the development of family ethics as part of a growing awareness towards the moral impact of relations and context in health care and social practice in general [54], and in end-of-life care in particular, as described in the introduction to the article. In line with this, Brown and Walter [55] argue that if end-of-life care shall be truly ‘holistic,’ the need to see dying patients as well as their relatives as “fully social beings, living, dying and caring within naturally occurring social networks” (p. 2378) is essential. Even if we have studied only nursing home patients and their close relatives, we find that both groups talk about a bigger circle of significant relatives and friends [56].

In reflecting on the empirical results, we find the ethics of authenticity, developed by Charles Taylor [57, 58] a fruitful theoretical position to broaden our understanding. Taylor discusses what makes a good life, and he points to how individuality plays a significant role. Individuality implies a strong belief in the human being, in reasoning, originality and diversity. A modern being is understood to be autonomous and in control. However, to live a good and authentic life, dialogue with others is fundamental. Taylor’s important point is that authenticity requires fellowship with others; to live an authentic life is to be dependent on something outside the individual person; what Taylor calls *horizons.* We think of families, family bonds, shared family stories and memories as a kind of horizon; something that can contribute to the patients’ identity and individuality. We suggest that paying attention to the patient as part of a family (as well as other networks), and involving and listening to families in planning for the future, can help us to see and understand what counts as important to the patient.

### Family ethics and paternalism

The literature shows that patients want their relatives to be informed and involved in decision making [36]. Patients are less likely to have ACP-conversations if the family is not involved [59], and some patients claim that their family members are the only people they want to talk to about dying and death, because these are found to be private, sensitive and intimate matters [60, 61]. Nevertheless, this is of course not the case for all patients, neither in nursing homes nor in health care in general. In interviewing 58 geriatric patients about their views on taking part in an ACP-conversation, Friis and Førde [36] found that 11 patients did not want the involvement of their families. Perhaps the reason for this is that patients worry that relatives’ views will override their own, a tendency described in dementia care [14]. Paternalism as part of health care is well known, and there are good reasons for paying attention to paternalism from family members as well. Proxy decisions are difficult, and can turn out to not be in line with the patient’s preferences [62]. We are aware that family relations can be difficult and even harmful to vulnerable elderly people, and families can feel the obligation to their family members as a heavy burden [13]. Conflicts in families also make decision processes difficult [63]. Even though there are families that fall short and fail in decision making processes, Verkerk et al. [13] describe most of them as good enough.

### The aim of ACP: factual outcomes and/or a family-centered process?

One of the results is that relatives helped to uncover what really mattered to the patients, but this ‘uncovering’ was not part of the observed ACP-conversations. What we see as important topics in the patients’ lives came to the surface during the interviews after the conversations were finished, like the loss of close relatives, moving from their private home to the nursing home, and not being able to live with a husband any more. An example is the case of Jenny who had recently moved to the nursing home and had never spoken to anyone about dying and death, not even her close sister who died a few months before. It is interesting to try to understand the differences between Jenny’s participation and short and reluctant answers in the admission conversation compared to how she participated in the interview. What is worth paying attention to is how Jenny became more talkative when we, during the interview, touched upon things that were important to her. The loss that Jenny had recently experienced and the fact that she had never spoken to anyone – not even her closest family – about dying and death are information and aspects that should be taken into consideration when questions of her own fragile future are on the agenda. Jenny had experienced death because she had lost people close to her. In an ACP-conversation, to ask about or refer to Jenny’s own recent experiences with dying and death may be a fruitful way to start talking about such sensitive issues. Here, relatives may play an important role in ACP-conversations because they will probably know about the patient’s losses and situations that could be connected to ACP-issues. Because of the niece’s knowledge about Jenny’s life, she asked questions that seemed to relate to Jenny in a very different way than the nursing home staff was able to, and by that she connects the past with the present.

Of course, what counts as significant issues in ACP is linked to what is seen as the aim of this process. ACP is described as a key to improve the experience of dying and death by enabling patients to consider end-of-life care options and preferences [64], but an ACP-discussion might also include the individual patient’s concerns and wishes [65]. We found that when the observed conversations turned to issues concerning the future and end-of-life care, the questions raised by the physician covered questions concerning medical treatment and/or hospitalization, where the patients seemed to struggle to answer clearly. What the patient’s concerns were was not on the agenda. While we agree that the medical and technological changes have made it necessary to consider future treatment options, also in nursing homes, we ask whether ACP-conversations could be improved by more attention being paid to ACP as a person-centered and a family-centered approach and process, rather than the factual outcomes [66]. Patients might find ACP more helpful if attention is payed to their individual needs [24], and family members can help to identify such needs and worries.

The value of paying more attention to how families matter is illuminated by how patients and families share experiences of the dying and death of others. Even if patients and relatives had never spoken openly together about their own future dying, they had many common experiences and memories, and some of these were talked about in some detail during the interviews. Including the patient’s and the relatives’ earlier experiences of dying and death in an ACP-conversation, can be a way of starting such a conversation, and can also contribute to understanding one’s own life and dying as part of a larger narrative, and thus something which brings meaning. Drawing on past experiences of the suffering and death of others can also be a way of talking about ‘good’ or ‘bad’ deaths. Garnett et al. [56] describe how confidants (relatives) tended to draw on their past experiences with illness and death when thinking about what might be acceptable when it came to questions concerning life-prolonging treatment, and the authors conclude that powerful past events affect relatives’ understanding and thinking.

### Strengths and limitations of the study

Observations and interviews in the nursing homes was found to be sufficient for obtaining valuable impressions about the way conversations were carried out. The aim of the present study was not to prove something, but to learn something by carefully studying and trying to understand existing practice [44, 67]. We find the data unique, original and important for planning and discussing future ACP practice in nursing homes. We are aware that the observations represent a short and temporary aspect of nursing home lives. If we had more inside experience from the nursing homes, and the number of nursing homes had been larger, our perception of the conversations might have been different or more varied. One limitation may be that relatives more often than patients took part in the interviews, and also more actively participated in the interviews than patients. Another limitation may be that the participating relatives may not be representative for relatives in general, but may be more positive, caring and responsible than others. With more participants, including patients without family or other next of kin, we could have gained more differentiated data.

## Conclusion

Analyses of how nursing home patients and relatives participated in ACP-conversations with nursing home staff, and what was expressed in interviews with the patients and relatives resulted in important findings. We found that patients and relatives can be perceived as intertwined units. The ways they were connected and how the relations were talked about, especially by the family members, confirm that family matters morally in an instrumental as well as an intrinsical way [13]. As a daughter or a niece, you are “given” a responsibility to care for your old mother or an aunt. None of the participants questioned this or talked about it as a heavy burden. Quite the opposite was seen in our study; that family relations and family responsibility seemed like valuable ends in themselves. We also found that if the aim of ACP is to get to know important issues and values to frail elderly patients, family members may play a very important role. This is because they know the patient in a different way than the staff; they know about the patient’s life story, and they were also able to make meaningful connections between the patients’ former life and the present situation. If ACP-conversations are carried out soon after admission to the nursing home, the involvement of family members may be even more essential. The third result indicates that to help health care professionals facilitate a conversation on future end-of-life care, a possible way to start is to ask the patient about earlier experiences and memories of loss and sorrow. These experiences are often part of a larger family story, and could be shared by the patient and relatives together. To be open to family ethics when planning for the patient’s future in the nursing home is to be open to diversity and nuances, and to the significance of the patient’s former life and experiences.

Results from this study have informed a guide on ACP in nursing homes (part 3 of the research project). In the guide, we suggest that patients must be informed that participation in ACP-conversations is voluntary, and they should be asked if they want relatives to participate. In the written invitation to ACP-conversations, the aims and content of the meeting is described, and we advise patients and relatives to discuss the future and possible wishes and preferences prior to the ACP-conversation. The guide suggests that an ACP-conversation should cover not only medical questions, but more broadly the life, future, and well-being of the elderly patient. Results on experiences with the guide will be published later. Future research is needed to further clarify how family ethics may be valuable in ACP in nursing homes as in other parts of health care. We suggest that ACP to a larger extent should be implemented in home care, followed by research with a particular focus on the role and impact of families who live with the patient on a daily basis. In teaching health care professionals about ethics theory which underpins ACP, in addition to autonomy, user involvement and shared decision-making, attention could also be given to family ethics.

## Abbreviations

ACP: Advance care planning
DNR: Do not resuscitate
